# Murine pulmonary responses after sub-chronic exposure to aluminum oxide-based nanowhiskers

**DOI:** 10.1186/1743-8977-9-22

**Published:** 2012-06-19

**Authors:** Andrea Adamcakova-Dodd, Larissa V Stebounova, Patrick T O’Shaughnessy, Jong Sung Kim, Vicki H Grassian, Peter S Thorne

**Affiliations:** 1 Department of Occupational and Environmental Health, University of Iowa, Iowa City, IA 52242, USA; 2 Department of Chemistry, University of Iowa, Iowa City, IA 52242, USA; 3 Interdisciplinary Graduate Program in Human Toxicology, University of Iowa, Iowa City, IA 52242, USA

**Keywords:** Aluminum, Nanowhiskers, High aspect ratio nanomaterial, Inhalation, Murine model, Pulmonary response, Toxicity

## Abstract

**Background:**

Aluminum oxide-based nanowhiskers (AO nanowhiskers) have been used in manufacturing processes as catalyst supports, flame retardants, adsorbents, or in ceramic, metal and plastic composite materials. They are classified as high aspect ratio nanomaterials. Our aim was to assess *in vivo* toxicity of inhaled AO nanowhisker aerosols.

**Methods:**

Primary dimensions of AO nanowhiskers specified by manufacturer were 2–4 nm x 2800 nm. The aluminum content found in this nanomaterial was 30% [mixed phase material containing Al(OH)_3_ and AlOOH]. Male mice (C57Bl/6 J) were exposed to AO nanowhiskers for 4 hrs/day, 5 days/wk for 2 or 4 wks in a dynamic whole body exposure chamber. The whiskers were aerosolized with an acoustical dry aerosol generator that included a grounded metal elutriator and a venturi aspirator to enhance deagglomeration. Average concentration of aerosol in the chamber was 3.3 ± 0.6 mg/m^3^ and the mobility diameter was 150 ± 1.6 nm. Both groups of mice (2 or 4 wks exposure) were necropsied immediately after the last exposure. Aluminum content in the lung, heart, liver, and spleen was determined. Pulmonary toxicity assessment was performed by evaluation of bronchoalveolar lavage (BAL) fluid (enumeration of total and differential cells, total protein, activity of lactate dehydrogenase [LDH] and cytokines), blood (total and differential cell counts), lung histopathology and pulmonary mechanics.

**Results:**

Following exposure, mean Al content of lungs was 0.25, 8.10 and 15.37 μg/g lung (dry wt) respectively for sham, 2 wk and 4 wk exposure groups. The number of total cells and macrophages in BAL fluid was 2-times higher in animals exposed for 2 wks and 6-times higher in mice exposed for 4 wks, compared to shams (*p* < 0.01, *p* < 0.001, respectively). However no neutrophilic inflammation in BAL fluid was found and neutrophils were below 1% in all groups. No significant differences were found in total protein, activity of LDH, or cytokines levels (IL-6, IFN-γ, MIP-1α, TNF-α, and MIP-2) between shams and exposed mice.

**Conclusions:**

Sub-chronic inhalation exposures to aluminum-oxide based nanowhiskers induced increased lung macrophages, but no inflammatory or toxic responses were observed.

## Background

Shape modification of nanomaterials, e.g., tailoring them to fiber-shaped nano-objects such as nanowires, nanowhiskers, nanobelts and nanotubes is especially attractive due to the unique properties that they acquire through these alterations [1]. However, the physical properties of the nanomaterials, such as shape or agglomeration state may play an important role in nanoparticle toxicity [2,3]. Recently, an increasing number of studies have focused attention on investigation of fiber-shaped or high aspect ratio nanomaterials (HARNs) due to their asbestos-like morphology and concern that these materials may have similar mechanisms of toxicity [4-10]. HARNs include materials such as carbon nanotubes (CNTs) that have been most studied thus far. According to Institute of Occupational Medicine (IOM, Edinburgh, Scotland), HARNs are classified “as part of the ‘one dimensional’ nanoscale building blocks; other one-dimensional particles include nanobelts and nanoribbons; these are nanostructures with high surface area and high aspect ratio (of greater than 10 to 1, length-to-diameter)” [7].

Key properties influencing pathogenicity of the fibers are fiber dimensions, geometry of the fibers, their biopersistence, high surface area, and surface reactivity [5,11]. It is well established that different types of asbestos fibers have unequal pathogenicity. In the case of asbestos fibers, a higher pathogenicity is associated with slower or no clearance, “frustrated phagocytosis” and thus higher biopersistence of fibers after exposure to long asbestos fibers as opposed to short ones [4]. In addition, the pathogenic potential of titanium dioxide (TiO_2_), that has been considered to be biologically inert for many years, was changed drastically when the shape of the material was modified [12]. Long micron-sized nanobelts of TiO_2_ (>15 μm) were shown to be highly toxic particles causing an inflammatory response by alveolar macrophages. Nanomaterials with higher aspect ratios have higher surface areas and thus increased chance of cell interactions. Due to increasing production of HARNs and subsequent increasing potential for inhalation exposure, attention should be paid to development of HARNs that are safe by design before the products are introduced to the market on a large scale [9,13].

Although many of these studies are focused on carbon nanotubes, our study is specifically focused on the toxic effects of commercially available aluminum oxide-based nanowhiskers. Nanowhiskers are single metallic high aspect ratio structures in the nanometer scale, also called nanowires or nanorods. Their diameter is in the nanometer range, however they can be more than a micron in length. Nanowhiskers have unique optical, chemical, and electrical properties. Many nanowhiskers are used for their hydrophobic characteristics. Thus, they find use in wrinkle-free, water or stain resistant clothing [14]. Aluminum oxide nanomaterials have been used as catalysts, catalyst supports, grain-growth inhibitors, heat-transfer fluids (suspensions), and polymer nanocomposites [15].

To date, not much research has been done on the behavior of metal oxide nanomaterials with a high aspect ratio in biological systems. The majority of studies have been conducted either *in vitro* or after instillation exposure, however inhalation is the most relevant route of exposure for evaluation of HARNs. Our study was designed to characterize pulmonary toxicity after inhalation exposure of AO nanowhisker aerosol. For generation of the aerosol we utilized a dry powder generation system developed by our group based on acoustical dry aerosol generator/elutriator [16,17], thus we eliminated a large number of possible interactions of nanomaterials with water or media.

## Materials and Methods

### Source of nanomaterial

Aluminum oxide nanowhiskers were purchased from Sigma-Aldrich (St. Louis, MO, USA). The primary dimensions of this material as reported by the manufacturer were 2–4 nm in diameter by 2800 nm in length. The nanowhiskers were used as received from the manufacturer.

### Particle characterization

#### Bulk characterization

Powder X-ray diffraction (XRD) was used to identify crystalline phases present in the sample. XRD was performed using a MiniFlex II X-ray diffractometer (Rigaku, Woodlands, TX, USA). Transmission electron microscopy (TEM) (JEOL JEM-1230, Tokyo, Japan) was used to measure the diameters of over 100 fibers to compare the average diameter to manufacturer’s specifications. A scanning electron microscope equipped with an energy dispersive spectrometer (SEM-EDS) (Hitachi S-3400 N, Tokyo, Japan) was used to image the nanowhisker aerosols passively collected on TEM grids in the whole body exposure chamber. TEM was also used to assess agglomerate sizes of the nanowhisker aerosols generated in the inhalation exposure chamber.

#### Surface characterization

Surface properties, surface area, and surface composition of the AO nanowhiskers were assessed. Surface area measurements were performed on an automated multipoint BET surface area apparatus (Quantachrome Nova 4200e, Boynton Beach, FL, USA) using nitrogen gas as the adsorbent. Samples were degassed at 300°C for 5 hrs under vacuum before the analysis. X-ray photoelectron spectroscopy (XPS) was used to probe the surface chemical composition characteristics of the sample (Ultra-Axis XPS system, Kratos, Manchester, UK).

#### Dissolution measurements

The dissolution of AO nanowhiskers was measured in artificial lysosomal fluid (ALF) (pH 4.5) and Gamble’s solution (pH 7.4) using inductively coupled plasma - optical emission spectroscopy (ICP-OES, Varian 720 ES, Walnut Creek, CA, USA). The ALF simulates the phagolysosomal composition and pH of alveolar and interstitial macrophages and the Gamble’s solution is used to mimic the interstitial fluid in the lungs [18]. Simulated biological fluids were prepared as previously described [19]. The nanowhisker solutions were incubated up to 4 wks at 38°C. The final solutions were filtered (0.2 μm, Millex-LG, Millipore, Billerica, MA, USA) and centrifuged for 30 min at 44,912 ×* g* in order to remove materials that were not dissolved, and analyzed by ICP-OES.

### Animals

Male C57Bl/6 J mice (6 wks old) were obtained from The Jackson Laboratory (Bar Harbor, ME, USA) and acclimatized for 12 days prior to exposures. They were housed in our vivarium in polypropylene, fiber-covered cages in HEPA-filtered Thoren caging units (Hazelton, PA, USA) in the Pulmonary Toxicology Facility at the University of Iowa during the study. Food (sterile Teklad 5% stock diet, Harlan, Madison, WI, USA) and water (*via* an automated watering system) was provided *ad libitum.* A 12-hr light–dark cycle was maintained in the animal room. The average body weight of mice at the end of acclimatization time was 23.7 ± 0.2 g. Mice were monitored during the whole study, weighed weekly and observed for any behavioral changes or clinical symptoms. All protocols used in this study were approved by the Institutional Animal Care and Use Committee and were compliant with NIH guidelines. At the time of necropsy the average body weights were 24.1 ± 0.2 g after 2-wk exposure and 25.0 ± 0.4 g after 4-wk exposure. Control mice (exposed to laboratory air) at the time of necropsy weighed 24.5 ± 0.4 g.

### Generation of dry aerosol and inhalation exposure of mice

**Figure 1:**
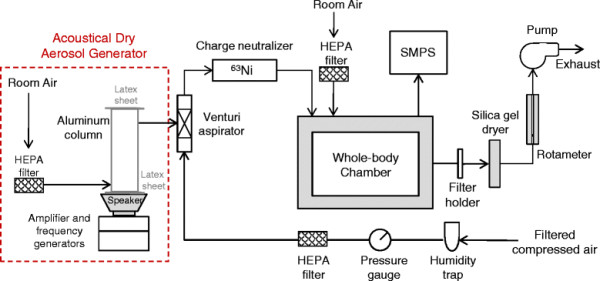
**Schematic of generation and exposure system used for AO nanowhiskers.** Acoustical dry aerosol generator/elutriator (ADAGE) was used to generate AO nanowhisker aerosol. Particle-laden air was pulled to the custom whole-body chamber through venturi aspirator and particle charge neutralizer.

The aerosol generation and exposure system is diagrammed in Figure 1. A dry aerosol of AO nanowhiskers was generated using an acoustical dry aerosol generator/elutriator (ADAGE) [16]. The ADAGE was adapted to include an electrically grounded aluminum elutriator (6.3 cm I.D. x 30.5 cm) to reduce aerosol losses within the column by minimizing static charge effects. Both ends of the aluminum column were covered with latex sheets that were stretched taut and held in place by rubber bands. The bottom of the column was placed over an 8-cm speaker. Speaker output was produced by adjusting an amplifier to produce an output of 7 V, and adjusting two frequency modulators to produce 10 Hz and 1 Hz overlapping signals. The ADAGE was also adapted with the addition of a venturi aspirator at the exit to enhance deagglomeration. The aspirator was designed as described by Cheng *et al.*[20] with a 1.5-mm nozzle diameter and operated such that a 14 L/min main flow rate of filtered, compressed air through the aspirator produced a 4 L/min suction flow rate. As shown in Figure 1, this suction air was pulled through a HEPA filter and into the ADAGE column *via* a port 2 cm from the bottom where it caused acoustically suspended AO nanowhiskers to exit a port 2 cm from the top and into the aspirator. The aerosol was transferred through a 20-mCi ^63^Ni source to remove static charge and into a 65 L dynamic whole-body exposure chamber [21]. Based on trials conducted before the animal exposures, loading 200 mg of AO nanowhisker powder on the bottom latex sheet of the column was sufficient to produce the desired aerosol concentration in the chamber (3.3 mg/m^3^) for 2 hrs. Thus, nanowhisker loading was repeated each 2 hrs during exposure. Furthermore, after adding a new bolus of powder the aerosol was made to bypass the chamber for 10 min to avoid administering a large spike in concentration observed during this time period. The size distribution of the generated dry aerosol during animal exposures was measured using a scanning mobility particle sizer (SMPS, TSI Inc., Shoreview, MN, USA, model 3071A electrostatic classifier and model 3010 condensation particle counter, measuring particle diameters in 105 size channels between 7.4 - 289 nm). To assure we were measuring the full range of particulates generated by our system, we also used a portable aerosol spectrometer (PAS, Grimm Technologies, Inc. Douglasville, GA, USA model 1.108, measuring particle diameters in 15 size channels between 0.3 – 20 μm). Combined measurements of SMPS and PAS showed that the majority of the particles were less than 300 nm in diameter therefore the subsequent size distribution was based on the SMPS measurements. 

The mass concentration of the aerosol in the whole-body chamber was measured gravimetrically every 2 hrs using 47-mm fiberglass filters (Whatman, Middlesex, UK) that were placed in stainless steel holders directly in line with the exhaust air flow of 24 L/min. Filters were weighed using a calibrated Mettler MT5 six-place balance (Mettler-Toledo, Inc., Columbus, OH, USA) placed in a dedicated climate-controlled room. Daily average concentrations ranged from 2.2 – 4.6 mg/m^3^.

Control animals were exposed in an adjacent laboratory to HEPA-filtered air in an identical whole-body exposure chamber and to a comparable sound pressure level (75–80 dB) that was produced during aerosol generation and exposure.

**Figure 2:**
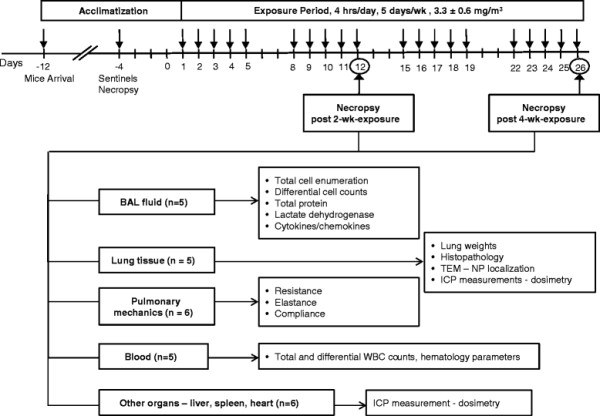
Experimental design for this inhalation study of AO nanowhiskers.

Mice were exposed in a sub-chronic experimental design for 4 hrs/day, 5 days/wk for 2 or 4 wks and necropsied within one hour after the last exposure (Figure 2). The average mass concentration of AO nanowiskers in the chamber was 3.3 ± 0.6 mg/m^3^.

### Bronchoalveolar lavage fluid and blood analyses

After the exposure, mice were euthanized with an overdose of isoflurane, cervical dislocation and exsanguination. The lungs from 5 mice were lavaged, *in situ*, three times with approximately 1 mL of sterile saline (0.9% sodium chloride solution, Baxter, Deerfield, IL, USA). The recovered fluid was centrifuged, the total white blood cells were counted using a hemocytometer and the supernatant was stored at −80°C for later analyses. For differential cell counts, resuspended cells in Hank’s balanced salt solution were spun (800 x g, 3 min, Cytospin 4, Thermo Shandon, Thermo Scientific, Waltham, MA, USA) onto microscope slides and air-dried. The slides were stained using HEMA 3 stain set, then 400 cells were counted to enumerate macrophages, neutrophils and lymphocytes. Total protein level and lactate dehydrogenase (LDH) activity in BAL fluid supernatants were determined using a Bradford protein assay (Bio-Rad Laboratories, Inc., Hercules, CA, USA) and a commercially available LDH kit (Roche Diagnostics, Penzberg, Germany). Multiplexed fluorescent bead-based immunoassays (Bio-Rad Laboratories, Inc., Hercules, CA, USA) were utilized to measure interleukin (IL)-6, IL-12(p40), tumor necrosis factor (TNF)-α, granulocyte macrophage colony stimulating factor (GM-CSF), keratinocyte-derived cytokine (KC), monocyte chemotactic protein (MCP)-1, and macrophage inflammatory protein (MIP)-1α, MIP-2 and interferon (IFN)-γ.

Blood samples were collected in EDTA-containing Multivette® tubes (SARSTEDT AG & Co., Nümbrecht, Germany) and analyzed using automatic hematology analyzer (Sysmex XT-2000i, Kobe, Japan).

### Elemental analysis in tissues and BAL fluid

Lung, heart, spleen and liver tissues from 6 mice in each group were stored at −80°C after removal. Tissues were lyophilized for approximately 16 hrs at 1.3 × 10^-1^ mBar and −50°C (Labconco Corp., Kansas City, MO, USA) and then weighed. The tissues were digested in a HotBlock™ digestion system (Environmental Express, Mt. Pleasant, SC, USA) at 95-98°C using mixtures of high purity concentrated nitric acid and hydrochloric acid (Optima grade, Fisher Scientific, Pittsburgh, PA, USA). Metal analysis was performed using ICP-OES (Varian 720 ES, Walnut Creek, CA, USA) with a method detection limit for Al of 0.0016 μg/g lung (dry wt). BAL fluid was centrifuged at 44,900 x *g* for 30 min and Al ion concentration in the supernatant was determined by inductively coupled plasma-mass spectrometry (ICP-MS, X Series, Thermo Scientific, Waltham, MA, USA).

### Pulmonary mechanics measurements

Pulmonary mechanics measurements were performed in mice exposed to AO nanowhiskers, sham-exposed mice as well as mice exposed to 3 U/kg bleomycin. The mice exposed to bleomycin by intratracheal instillation served as positive controls [22] and were necropsied 21 days post exposure.

To measure pulmonary mechanics, mice were administered 90 mg/kg of pentobarbital sodium (Ovation Pharmaceuticals, Inc. Deerfield, IL, USA) by intraperitoneal injection (n = 5 mice) followed by tracheotomy using a tracheal cannula with luer adapter (1.3 mm, length 20 mm, Harvard Apparatus, Holliston, MA, USA). Mice were then connected to a computer-controlled small animal ventilator (flexiVent, SCIREQ, Montreal, QC, Canada) set at a frequency of 150 breaths/min, a tidal volume of 10 mL/kg and a positive end-expiratory pressure of 2 – 3 cm H_2_O. The forced oscillation technique was used to assess Dynamic Resistance (R, the level of constriction in the lungs) and Dynamic Compliance (C, the ease with which the lungs can be extended) measurements. From these two measurements Dynamic Elastance (E) (the inverse of the Compliance, E = 1/C) was calculated by flexiVent software (version 5). These measurements R, C, and E represent parameters of the whole respiratory system (airways, lung and chest wall). To measure airway hyper‐reactivity, mice were challenged with increasing methacholine aerosols (1, 3, 10 and 30 mg/mL), generated with an in-line nebulizer (10 sec) directly through the canulated trachea. Response to methacholine was determined every 30 sec for 5 min, ensuring the measurement parameters stabilized. The lungs were inflated to total lung capacity after each methacholine dose, to prevent atelectasis and to return the measurements back to baseline.

### Histopathology of lung tissues

A subset of mice (n = 5 per group) were not lavaged but were perfused through the heart with saline and their lungs were fixed using 10% Zn formalin. The lungs were then embedded in paraffin blocks, sectioned at 5 μm and stained with hematoxylin and eosin (H & E). Light field microscopy was used to evaluate stained sections of lung tissues with the focus on presence or absence of inflammatory cells infiltrates, lymphoid agglomerates or fibrosis.

### Statistical analyses

Data from controls and AO nanowhisker-exposed mice were compared using *t*-tests for equal and unequal variance. A *p*-value less than 0.05 was considered significant. Area under the curve (AUC) was calculated for pulmonary mechanics measurements and R, C and E were compared using analyses of variance (ANOVA) for repeated measures (SAS Ver. 9.2, SAS Institute Inc., Carry, NC, USA).

## Results

### Nanowhisker characterization

**Figure 3:**
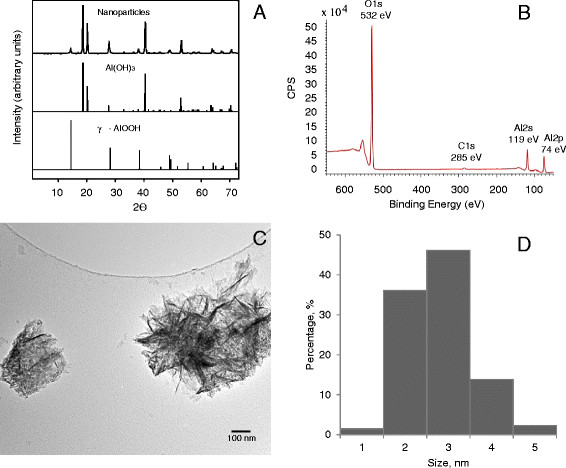
**Characterization of the AO nanowhiskers.** XRD patterns of the nanowhiskers compared to XRD patterns for two reference materials, bayerite and boehmite (**A**), X-ray photoelectron spectrum of the nanowhiskers (**B**), TEM image of AO nanowhiskers deposited from methanol onto TEM grid (**C**), and size distribution of nanowhisker diameters (**D**).

Crystalline phases present in the AO nanowhiskers were determined from powder XRD. These data are shown in Figure 3A. The manufacturer’s stated formula for the nanowhiskers is Al_2_O_3_. Attempts to match the XRD pattern of the nanowhiskers with Al_2_O_3_ phases were unsuccessful, however, the XRD pattern of the AO nanowhiskers aligned well with XRD patterns of aluminum hydroxide, Al(OH)_3_, and aluminum oxyhydroxide, γ-AlOOH. Based on the XRD patterns, it appears that the aluminum oxide nanowhiskers are a mixture of bayerite (Al(OH)_3_) and boehmite (AlOOH) [23]. Additionally, it was determined by ICP-OES analysis that there is about 30% Al in these nanowhiskers by mass. 

Surface composition examination using XPS (Figure 3B) showed binding energy peaks in the Al2p, Al2s, C1s, and O1s regions of the photoelectron spectrum with values of 74, 119, 285, and 532 eV, respectively. The Al2p peak at 74 eV lies within the binding energy region for aluminum oxides and hydroxides, where the hydroxides have peaks close to 74 eV [24]. The O1s peak position at 532 eV is indicative of surface hydroxyl groups, Al-OH [25]. The peak at 285.0 eV is due to adventitious carbon on the nanowhiskers surface, a common feature seen for surfaces exposed to the ambient environment.

Figure 3C shows a TEM image of agglomerated nanowhiskers deposited on a TEM grid from a methanol solution. The diameters of individual fibers were measured to obtain a size distribution. As shown in Figure 3D, the size distribution is narrow with an average diameter of 3 ± 0.6 nm based on the measurement of over 100 fibers. This result agrees well with the manufacturer’s specified diameter of 2–4 nm. The length of the nanowhiskers could not be measured precisely because the nanowhiskers were tangled as can be seen in Figure 3C. Although the length of whiskers reported by the manufacturer to be 2800 nm, they appeared to range from 50 to 300 nm. The specific surface area of the sample was calculated in the nitrogen relative pressure range from 0.05 to 0.1 where the BET isotherm is most linear. This yielded a surface area of 320 ± 4 m^2^/g for these nanowhiskers. Physicochemical properties of AO nanowhiskers and the generated aerosol are summarized in Table 1.

**Table 1:** Summary of physicochemical properties of AO nanowhiskers and generated aerosol

	**AO nanowhiskers**
Structure	Crystalline
Phases	Al(OH)_3_ and γ-AlOOH
Primary diameter^1^	3 ± 0.6 nm
Length^1^	~ 50 – 300 nm
Surface functionalization	O, O-H and H_2_O
BET surface area	320 ± 4 m^2^/g
Aerosol size distribution in whole-body exposure chamber^2^	GM = 150 nm GSD = 1.6
Exposure concentration	3.3 ± 0.6 mg/m^3^

^1^ Primary diameter and length as measured by TEM in our laboratory. The length of the nanowhiskers could not be measured precisely since they appeared as tangled bundles. The length reported by manufacturer was 2800 nm.

^2^ Geometric mean mobility diameter (GM) and geometric standard distribution(GSD).

### Dissolution in artificial lung fluids

**Figure 4:**
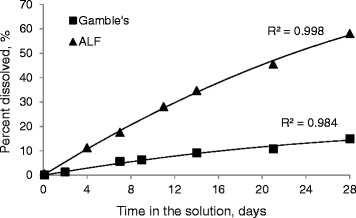
Dissolution kinetics of AO nanowhiskers in simulated biological fluids.

The propensity of AO nanowhiskers to dissolve into ions was investigated in solutions designed to represent dissolution in the airway surface liquid or in the macrophage phagolysosome. ALF with a relatively low pH of 4.5 contains a large amount of citric acid that is supposed to simulate the role of proteins produced by macrophages to dissolve foreign materials. Gamble’s solution with the pH of 7.4, simulates the pH environment found in the interstitial fluid in the lungs. As can be seen from Figure 4, 9% of AO nanowhiskers were dissolved in Gamble’s solution after 2 wks and about 15% after 4 wks. A linear release of aluminum was observed during dissolution in ALF buffer, where almost 35% of nanowhiskers were dissolved in 2 wks and about 58% in 4 wks.

### Aerosol characterization

**Figure 5:**
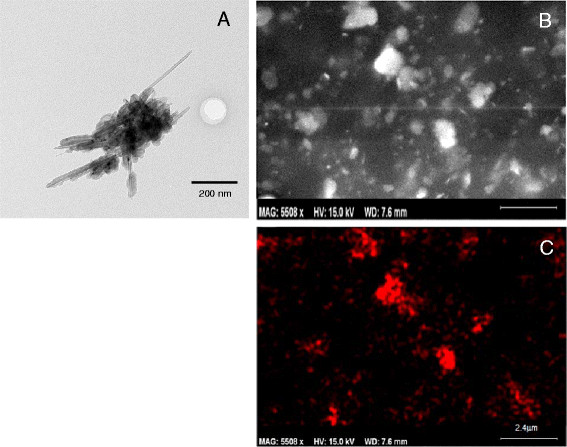
**AO nanowhiskers collected in the exposure chamber.** A TEM image (**A**), SEM image (**B**) and SEM EDS map (**C**) of the same region as shown in Figure 5B. Scale bars in 5B and 5 C represent 2.4 μm. Red color represents signal collected at characteristic X-ray energy for Al metal.

**Figure 6:**
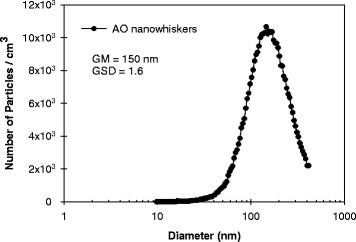
**Particle size distribution of AO nanowhisker aerosols generated by ADAGE to the whole-body exposure chamber.** The geometric mean mobility diameter(GM) of aerosolized AO nanowhiskers was 150 nm with geometric standard deviation (GSD) 1.6. Measurement was performed using SMPS sampling exhaust flow from the chamber.

AO nanowhisker aerosols collected in the exposure chamber were imaged using TEM and SEM (Figure 5A and 5B). Figure 5A is a TEM image of a nanowhisker agglomerate with protruding whiskers. SEM-EDS analysis (Figure 5C) of the agglomerates detected Al at its characteristic X-ray energy within the aerosols. The size distribution of the nanowhisker agglomerates comprising the aerosol in the exposure chamber had a geometric mean mobility diameter (GM) of 150 nm and a geometric standard deviation (GSD) of 1.6 (Figure 6).

### Dosimetry of AO nanowhiskers in the tissues and BAL fluids

Retained Al in the lung tissues was determined by ICP-OES (Table 2). The mass of Al in sham-exposed mice was very low (0.25 ± 0.17 μg/g lung (dry wt)). Both groups of mice exposed to AO nanowhiskers for 2 or 4 wks had significantly higher (*p* < 0.001) mass of Al in the lung tissues. The mass of Al in lung tissues from mice exposed for 2 wks and 4 wks was 8.10 ± 0.79 and 15.37 ± 0.31 μg/g lung (dry wt), respectively. These values correspond to 0.24 μg Al/mouse for 2 wk-exposed and 0.51 μg Al/mouse for 4-wk exposed animals.

**Table 2:** Al concentration in lung tissue of shams and AO nanowhisker-exposed mice determined by ICP-OES

**Al concentration**	**Shams**	**Exposure to AO nanowhiskers for:**	
		**2 wks**	**4 wks**
Lung tissue, μg/g lung (dry weight)	0.25 ± 0.17	8.10 ± 0.79***	15.37 ± 0.31***

Values are expressed as mean and standard error; ***p < 0.001 significantly different from shams.

We estimated the deposited dose as a function of minute volume of 25 mL/min, an aerosol concentration of 3.3 mg/m^3^ and a deposition coefficient of 0.10 in the tracheo-bronchiolar and pulmonary region [26,27]. Using this calculation and considering that the AO nanowhiskers were 30% Al by XRD and ICP-OES, we estimated that 5.88 μg Al/mouse was delivered to the lungs after exposure to AO nanowhiskers for 2 wks and 11.76 μg Al/mouse after 4 wk exposure. Our estimation did not take into the consideration clearance of particulates *via* the mucociliary escalator to the trachea or gastrointestinal tract, clearance of particulates to lymphatic system, nor translocation of nanomaterial to other organs. We found that there was 4% of retained Al in the lungs from our estimated Al dose.

We determined the levels of Al ions in particle- and cell-free BAL fluid by ICP-MS, however the amounts of Al^3+^ were very low and not different between shams and exposed groups (0.55 μg/L ± 0.40, 0.67 μg/L ± 0.61 and 0.70 μg/L ± 0.28 in shams and in mice exposed for 2 wks and 4 wks, respectively). These results show that little AO dissolved in lung fluids. The levels of Al in hearts, spleens and livers were not significantly different between AO-nanowhisker-exposed groups and shams.

### Evaluation of BAL fluid and hematology parameters

**Figure 7:**
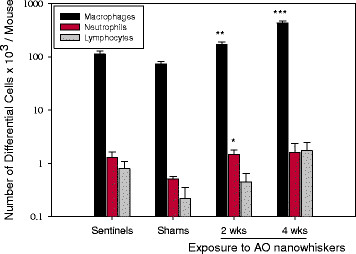
**Number of differential cells in BAL fluid in sentinels, mice exposed to laboratory air (shams) or mice exposed to AO nanowhiksers for 4 hrs/day for 2 or 4 wks (at daily concentration of 3.3 mg/m**^2^**);** Data are expressed as mean ± SE; *p < 0.05, **p < 0.01, ***p < 0.001 significantly different from shams.

As shown in Table 3, exposure to dry aerosols of AO nanowhisker for 2 or 4 wks caused significantly higher (*p* < 0.01 and *p* < 0.001, respectively) recruitment of total cells in BAL fluid (171.6 ± 19.7 x 10^3^ and 440.3 ± 33.6 x 10^3^ cells/mouse, respectively) as compared to control mice (74.0 x 10^3^ ± 7.9 cells/mouse). This elevation was represented mainly by significant increases of macrophages in BAL fluid in animals exposed to the AO nanowhiskers for 2 wks (2-fold increase) or 4 wks (6-fold increase) in comparison to controls (Figure 7). The number of neutrophils in BAL fluid per mouse was significantly increased in AO nanowhisker-exposed animals for 2 wks (1.5 x 10^3^ ± 0.3) compared to sham-exposed mice however this increase was not biologically meaningful. Such numbers of neutrophils in BAL fluid are often seen in controls or naïve mice (sentinels).

**Table 3:** Total number of cells, concentration of total protein and activity of LDH in BAL fluid in shams and AO nanowhisker-exposed mice

**Parameter**	**Shams**	**Exposure to AO nanowhiskers for:**	
		**2 wks**	**4 wks**
Total cells/mouse, n x 10^3^	74.0 ± 7.9	171.6 ± 19.7 **	440.3 ± 33.6 ***
Total protein, μg/mL	107.7 ± 4.7	108.0 ± 1.5	101.2 ± 2.7
LDH activity, U/L	180 ± 18	146 ± 17	191 ± 10

Values are expressed as mean and standard error; *p < 0.05, **p < 0.01, ***p < 0.001 significantly different from sham-exposed animals.

Values of total protein as well as LDH activity in BAL fluid were normal and not elevated in comparison to controls (Table 3). Similarly, none of the measured cytokines (IL-6, IL-12(p40), TNF-α, GM-CSF, KC, MCP-1, MIP-1α, IFN-γ and MIP-2) were different from controls. Most of measured cytokines were below the limits of detection, except IL-12(p40), but the concentrations were not significantly different between air- and AO nanowhisker-exposed groups for 2 or 4 wks (38.2 ± 3.5, 37.2 ± 2.3, and 34.7 ± 1.2, respectively).

The total number of white blood cells, red blood cells, neutrophils, lymphocytes, monocytes, eosinophils, basophils, and values of hemoglobin and hematocrit were not significantly different between controls and AO nanowhisker-exposed mice (Table 4).

**Table 4:** Blood parameters in shams and mice exposed to AO nanowhiskers for 2 or 4 wks

**Whole Blood Parameter**	**Shams**	**Exposure to AO nanowhiskers for:**	
		**2 wks**	**4 wks**
White blood cells × 10^3^/μL	3.09 ± 0.67	3.78 ± 0.86	3.39 ± 1.02
Red blood cells × 10^6^/μL	11.16 ± 0.31	10.48 ± 0.36	10.48 ± 0.44
Hemoglobin, g/dL	17.10 ± 0.45	16.33 ± 0.59	16.67 ± 0.49
Hematocrit, %	54.51 ± 1.22	50.96 ± 1.87	50.88 ± 1.61
Neutrophils × 10^3^/μL	0.72 ± 0.30	0.55 ± 0.10	0.55 ± 0.19
Lymphocytes × 10^3^/μL	2.05 ± 0.37	2.76 ± 0.71	2.46 ± 0.73
Monocytes × 10^3^/μL	0.28 ± 0.05	0.38 ± 0.08	0.32 ± 0.10
Eosinophils × 10^3^/μL	0.04 ± 0.01	0.09 ± 0.03	0.06 ± 0.02
Basophils × 10^3^/μL	0.009 ± 0.004	0.006 ± 0.004	0.001 ± 0.001

### Lung histopathology and pulmonary mechanics measurements

**Figure 8:**
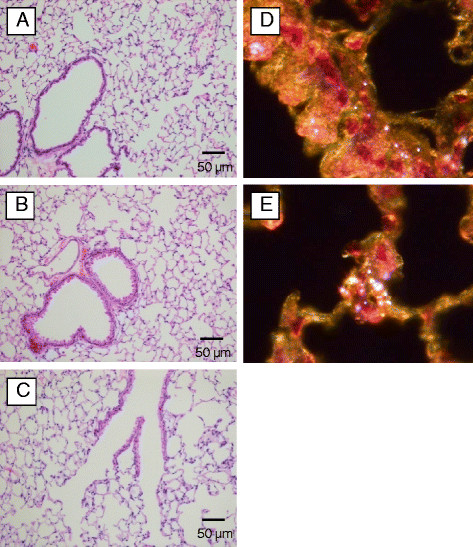
**Microslides of lung tissue with H&E staining from control mice (A), mice exposed to AO nanowhiskers for 2 (B) or 4 wks (C).** Figures **D** and **E** show localization of particulates in lung epithelium and macrophages in the lungs from mice exposed to AO nanowhiskers (dark field images, magnification 100 ×).

**Figure 9:**
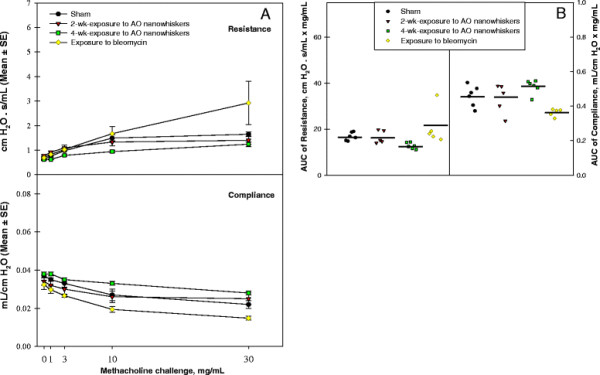
**Measurement of pulmonary mechanics in mice exposed to AO nanowhiskers.** Pulmonary mechanics measurements expressed as resistance and compliance in sham- and AO nanowhisker-exposed mice after increasing concentration of methacholine (1, 3, 10 and 30 mg/mL). The mean and SE of resistance and compliance versus methacholine concentrations (**A**). The individual and mean area under the curve (AUC) versus all increasing methacholine concentrations (**B**).

Histopathology evaluation of lung tissues stained with H&E did not reveal any signs of inflammatory cells infiltrates, lymphoid agglomerates, or fibrosis. All lung tissues from control mice or mice exposed to AO nanowhiskers for 2 or 4 wks were evaluated as normal (Figure 8). These results were consistent with pulmonary mechanics parameters such as resistance, elastance and compliance. All these parameters were assessed in mice challenged with increasing doses of methacholine aerosol. Comparison of means of AUC between controls and AO nanowhiskers-exposed mice using ANOVA for repeated measures showed no significant differences (Figure 9A and 9B). Thus, inhalation exposure to AO nanowhiskers did not produce clinically significant remodeling of the airway or airway hyperreactivity of the mice. On the other hand, mice exposed to the positive control, bleomycin, developed fibrotic changes and significantly increased pulmonary resistance and decreased compliance measured after methacholine challenge as compared to controls (Figure 9A and B).

## Discussion

Our study is one of few to use a dry powder generation system for inhalation exposure to HARNs. Generation and exposure systems utilizing dry aerosols are the best options to recreate human exposure conditions, since there have been many challenges with characterization of nanomaterials in different *milieu* (e.g. water or culture media). These challenges are even more pronounced for HARNs mainly due to the higher aspect ratio of fibers in comparison to other nanomaterials as well as increased surface area that can lead to higher surface interactions than other nanomaterials [7].

The Al^3+^ concentration detected in the lungs of mice after 4 wk exposure to Al nanowhiskers was 0.505 μg Al/mouse. Considering that 30% of AO nanowhisker mass is Al, the mouse burden of AO nanowhiskers would be 1.68 μg AO nanowhiskers/mouse, which corresponds to the lung burden accumulated by a 70 kg person exposed to a concentration of 1 mg/m^3^ for 44 hrs (assuming breathing 15 breaths/min, 600 mL/breath, and a pulmonary deposition fraction for 150 nm particles of 0.2 [27] and delivered AO nanowhisker dose to human 4712 μg). Extrapolating mass of AO nanowhiskers per alveolar epithelial surface area for mouse (0.05 m^2^) and the human (102 m^2^) [28], the mouse burden would be 34 μg/m^2^ and human burden would be 46 μg/m^2^.

The nanowhisker aerosol produced by the novel generation process applied during this study was characterized by an SMPS and TEM. Those results suggest that this dry-dust generation method was capable of producing a size distribution comparable to those produced in our previous studies [18,29-32] in which a powder suspension was nebulized and the droplets dried prior to entry into the chamber. Our previous efforts to use sonic energy to disperse nanopowders were not as successful when applied to other nanopowder types and without the alterations applied during this study [33]. The addition of the venturi aspirator and static-charge grounding strap to an aluminum (rather than plastic) elutriator were adaptations to the ADAGE that resulted in a consistent output of nanowhisker agglomerates with a GM of 150 nm.

We attempted to characterize the particles in the lungs after exposure using bright and dark field microscopy as well as TEM-EDS. However, we were unable to locate particles in lung tissue using TEM. This might be attributed to low retained doses of AO nanowhiskers in the lungs and low amount of Al in the AO nanowhiskers, thus it is possible that Al ion concentrations in the lung tissues were too low to produce a signal in TEM‐EDS.

As with our previously studied materials [18,29,31], characterization of the nanomaterials confirmed that characterization data provided by manufacturers needs to be independently determined due to batch to batch variability and the fact these materials are not always characterized using state-of-the-art instrumentation. Based on the XRD patterns, it appears that AO nanowhiskers were not a pure aluminum oxide phase such as Al_2_O_3_ but instead were a mixture of bayerite (Al(OH)_3_) and boehmite (AlOOH) [23]. Thus, complete and independent characterization of material used for toxicity evaluation is essential [23].

Many recent studies have related bioavailability and subsequent toxicity of nanomaterials to the ability of the nanomaterial delivered to biological or environmental milieu to release soluble metal ions to proximate cells or organisms [34-36]. Our previous study found this association between nanoparticle toxicity and dissolution [18]. However, with AO nanowhiskers, we found that 35% of nanowhiskers were dissolved in ALF buffer (pH 4.5) in 2 wks and 58% in 4 wks. About 15% of AO nanowhiskers dissolved in Gamble’s solution (pH 7.4) which represents extracellular fluid (Figure 4) in 4 wks. We found that it was difficult to dissolve AO nanowhiskers even in concentrated nitric acid. The observation of relatively high dissolution of AO nanowhiskers in ALF but low dissolution *in vivo* suggests that AO nanowhiskers undergo ligand-promoted dissolution in the simulated biological buffers due to the presence of citric acid [37].

In ligand–promoted dissolution, ligands form surface complexes that can polarize metal-oxygen bonds, thus facilitating the detachment of metal species from the surface. Ligands are capable of forming surface chelates, *i.e.,* bi- or multi-dentate ligands such as oxalate or citrate, can facilitate these interactions and enhance the dissolution of aluminum oxide [38]. It has been shown that citrate adsorbs to aluminum oxyhydroxide surface in a predominantly inner-sphere manner which facilitates the dissolution of this mineral [39]. Citrate is a molecule of both environmental and biological importance since it is present in many naturally-occurring systems and in blood plasma at a concentration of 0.1 mM [40,41]. It is used in ALF solution to represent protein binding to foreign objects. Here the interaction of citrate with AO nanowhiskers plays an important role in the nanomaterial dissolution. The solutions of artificial biological fluids are only facsimiles of actual lung fluids and some AO nanowhiskers were likely cleared from the pulmonary system after exposure. Thus, it is most likely that the nanomaterials were not present in lung fluids for as long as they were present in our simulated lung fluids for our dissolution studies (2 or 4 wks). Low solubility of AO nanowhiskers in epithelial lining fluid was also confirmed by very low levels of Al ions in supernatants of BAL fluid measured by ICP-MS.

There was significantly higher recruitment of alveolar macrophages in BAL fluid with longer exposure to AO nanowhiskers. However, the number of neutrophils and lymphocytes were not elevated meaningfully. Thus, it seemed that macrophages were able to control the aerosol load in the pulmonary system without upregulation of cytokine production or cytotoxicity. The experimental design with the total exposure period lasting up to 28 days would be sufficient for the mice to develop fibrosis in the lung tissues, however our histopathology evaluation of the lung tissues as well as pulmonary mechanics assessment found no pathological changes. This may be attributed to the low fibrogenicity of the material or low lung burden of AO nanowhisker in exposed mice. These findings are consistent with our measurements of total protein, LDH activity as well as cytokines/chemokines measured in BAL fluid. We also analyzed basic hematology parameters in the blood for possible systemic effects of inhaled aerosol of AO nanowhiskers, but did not find any deviations from our control animals.

A rat inhalation study of AlOOH with primary particle range of 10 – 40 nm (with mass median aerodynamic diameter (MMAD) of agglomerated particles in inhalation chambers 1.7 and 0.6 μm, respectively) for 4 wks (6 hrs/day, 5 days/wk), found significant pathology changes in BAL parameters and lung pathology only at the highest exposure concentration (28 mg/m^3^), but not at concentrations of 0.4 and 3 mg/m^3^[42]. Despite a number of differences between Pauluhn and our study (e.g. different animal models, morphology and size of nanomaterials, and length of exposures) their findings are noteworthy. Similarly, like our study, this study did not find any substantial extrapulmonary translocation of Al.

In our presented study, the estimated lung burden of AO nanowhiskers per mouse after 2-wk-exposure was 19.6 μg/mouse and after 4-wk-exposure it was 39.2 μg/mouse. We observed minimal pulmonary responses after this exposure similarly as after sub-acute (2-wk) exposure to silver (Ag) NPs with estimated lung burden of 29.4 μg/mouse [31]. On the other hand, in our previous study of copper (Cu) NPs [32] we observed substantial neutrophilic response after sub-acute exposure to Cu NPs with estimated lung burden of 32 μg/mouse. Certainly, there are many physicochemical characteristics of nanomaterials that play role in their toxicity (e.g. bulk and surface chemical compositions, size, phase, propensity for dissolution, ability for clearance or translocation etc.) and they need to be taken into the consideration when these materials are evaluated.

Our inhalation exposure to AO nanowhiskers did not result in “frustrated phagocytosis” and subsequent inflammation, which might have been expected since shorter fibers are more likely to be phagocytosed by alveolar macrophages than longer fibers. Another reason might be that the AO nanowhiskers after generation to the exposure chamber appeared more like tangled bundles of materials rather than individual fibers. And thus it is possible that they behaved more like particles that could be cleared from respiratory system by the mucociliary escalator. It appears that alveolar macrophages in our study were not overloaded since we did not observe an increased production of pro-inflammatory cytokines/chemokines and subsequent higher recruitment of neutrophils that would otherwise lead to pathologic outcomes. Furthermore, AO nanowhiskers may have endured some other changes after entering the respiratory system, such as breakdown that might further increase their clearance from pulmonary system.

A number of studies report that surface area of particles is associated with the magnitude of airway inflammation [43-45]. The surface area of AO nanowhiskers (320 ± 4 m^2^/g) was the largest of all nanomaterials studied thus far by our group [18,29-32]. Therefore and also considering current knowledge about HARNs concerning their toxicity, we hypothesized that there might be a potential for a higher toxicity of this nanomaterial. However, as in our previous study with two TiO_2_ nanoparticles with different surface areas [30] as well as other studies [46,47], our results add the evidence that there are many physicochemical properties of nanomaterials that play a role in toxicity. Larger surface area of nanomaterials is only one consideration and is not always responsible for higher toxicity. Other studies have shown that the shape of fibrous material has a decisive role in their toxicity, however AO nanowhiskers that we investigated exhibited low toxicity in this study. Cell surface injury was observed in macrophages exposed to fibrous TiO_2_, but no significant changes in macrophages were found in case of particulate TiO_2_[48]. *In vitro* study of carbon nanotubes and nanofibers found that long, straight, well-dispersed nanofilaments caused significantly higher production of TNF-α and reactive oxygen species (ROS) in human peripheral blood mononuclear cells than highly curved or tangled materials [49].

## Conclusions

A murine sub-chronic inhalation exposure consisting of a dry aerosol of aluminum-oxide based nanowhiskers caused increased recruitment of macrophages to the lungs, but no inflammatory or toxic responses. It appears that there was sufficient pulmonary clearance and AO nanowhiskers did not cause inflammatory, cytotoxic or immune responses, lung pathology, or changes in pulmonary function in exposed mice. Considering primary dimensions of studied material, large surface area as well as low solubility in intracellular or extracellular simulated biological fluids, one would predict higher biopersistence of the material in the lungs with subsequent toxicity similar to fibrous materials. However, the mass of AO nanowhiskers retained in the lung represented only about 4% of our estimated dose, thus we presume that AO nanowhiskers were readily phagocytized by macrophages probably because they were presented more as tangled bundles rather than more crystalline fibrous materials or that the deposition coefficient was substantially lower than the assumed 0.10.

## Abbreviations

ADAGE: Acoustical dry aerosol generator/elutriator;Ag: Silver;ALF: Artificial lysosomal fluid;Al(OH)_3_: Aluminum hydroxide;γ-AlOOH: Aluminum oxyhydroxide;γ-AlOOH: Boehmite;ANOVA: Analyses of variance;AO nanowhiskers: Aluminum oxide-based nanowhiskers;AUC: Area under the curve;BAL: Bronchoalveolar lavage;C: Dynamic compliance;CNTs: Carbon nanotubes;Cu: Copper;E: Dynamic Elastance (E);HARNs: High aspect ratio nanomaterials;H & E: Hematoxylin and eosin;GM: Geometric mean;GM-CSF: Granulocyte macrophage colony stimulating factor;GSD: Geometric standard deviation;ICP-MS: Inductively coupled plasma-mass spectrometry;ICP-OES: Inductively coupled plasma - optical emission spectroscopy;IL: Interleukin;IFN: Interferon;KC: Keratinocyte-derived cytokine; LDH: Lactate dehydrogenase; MCP: Monocyte chemotactic protein; MIP: Macrophage inflammatory protein;MMAD: Mass median aerodynamic diameter; PAS: Portable aerosol spectrometer; R: Dynamic resistance; SEM-EDS: Scanning electron microscope equipped with energy dispersive spectrometer;SMPS: Scanning mobility particle sizer; TEM: Transmission electron microscopy; TiO_2_: Titanium dioxide; TNF: Tumor necrosis factor; XPS: X-ray photoelectron spectroscopy; XRD: Powder X-ray diffraction.

## Competing interest

Vicki H. Grassian holds stock in Nanoscale Corp. (Manhattan, KS, USA).

## Authors' contributions

AAD participated in the study design, conducted the animal exposure studies, biological assays, pulmonary mechanics measurements, data analysis, and drafted the manuscript; LVS performed nanomaterial characterization, dissolution studies, elemental analyses, and drafted parts of the manuscript. PTO designed the acoustical dry aerosol generator, set up and coordinated aerosol generation system for inhalation exposures; characterized exposure concentrations and aerosol size distribution. JSK participated in the study design, conduction of animal studies, biological assays and elemental analyses; VHG initiated nanomaterial shape dependent studies and the integrated study design as well as led analyses of nanomaterial characterization and dissolution data and edited the manuscript; PST designed the acoustical dry aerosol generator, designed the animal study, coordinated biological assays and data analysis, and authored the final manuscript. All authors read and approved the final manuscript.
